# Social support and the effects of the COVID-19 pandemic among a cohort of people living with HIV (PLWH) in Western Kenya

**DOI:** 10.1371/journal.pgph.0000778

**Published:** 2023-02-28

**Authors:** Adel Mburia-Mwalili, Karla D. Wagner, Edith Kamaru Kwobah, Lukoye Atwoli, Maurice Aluda, Brianna Simmons, Jayne Lewis-Kulzer, Suzanne Goodrich, Kara Wools-Kaloustian, Jennifer L. Syvertsen

**Affiliations:** 1 School of Public Health, University of Nevada, Reno, Nevada, United States of America; 2 Mental Health Department, Moi Teaching and Referral Hospital, Eldoret, Kenya; 3 Department of Mental Health and Behavioural Sciences, Moi University School of Medicine, Eldoret, Kenya; 4 Department of Medicine, Brain and Mind Institute, Medical College East Africa, The Aga Khan University, Nairobi, Kenya; 5 Kenya Medical Research Institute (KEMRI), Kisumu, Kenya; 6 Department of Anthropology, University of California, Riverside, California, United States of America; 7 Department of Obstetrics, Gynecology & Reproductive Sciences, Bixby Center for Global Reproductive Health, University of California, San Francisco, California, United States of America; 8 Department of Medicine, School of Medicine, Indiana University, Indianapolis, Indiana, United States of America; University of California Irvine, UNITED STATES

## Abstract

As coronavirus disease (COVID-19) was declared a pandemic in 2020, countries around the world implemented various prevention strategies, such as banning of public and social gatherings, restriction in movement, etc. These efforts may have had a deleterious effect on already vulnerable populations, including people living with HIV (PLWH). PLWH were concerned about contracting COVID-19, the impact of COVID-19 on their social networks that provide social support, and the continued availability of antiretroviral medications during the pandemic. In addition, their mental health may have been exacerbated by the pandemic. The purpose of this study was to explore pandemic-related concerns among a cohort of PLWH in Kenya and investigate social support factors associated with symptoms of depression and anxiety. This study is part of a larger cohort study that recruited from two clinics in Western Kenya. Data are drawn from 130 PLWH who participated in two phone surveys about experiences during the pandemic in 2020 and 2021. Participants reported a variety of concerns over the course of the pandemic and we documented statistically significant increases in symptoms of depression and anxiety over time, which affected some participants’ ability to adhere to their antiretroviral medication. However, a small but statistically significant group of participants reached out to expand their networks and mobilize support in the context of experiencing mental health and adherence challenges, speaking to the importance of social support as a coping strategy during times of stress. Our findings call for holistic approaches to HIV care that consider the broader political, economic, and social contexts that shape its effectiveness.

## Introduction

The World Health Organization declared severe acute respiratory syndrome coronavirus-2 (SARS-CoV-2), which causes coronavirus disease (COVID-19), a pandemic on March 11, 2020 [1]. The first case of COVID-19 was reported in Kenya on March 13, 2020 [2] and as of December 31, 2021, there were 295,028 total confirmed COVID-19 cases and 5,378 reported deaths in Kenya [3]. At the beginning of the first wave in 2020, as in other countries around the world, Kenya implemented several public health measures to prevent and control the spread of COVID-19, including banning public gatherings, curfews, closure of all schools, restaurants, and bars, and restriction of movement into and from counties deemed hotspots of COVID-19 [4–6]. As subsequent variants of COVID-19 emerged, Kenya continued to implement some of these measures in different parts of the country, based on emerging regional case trends, to prevent further spread of COVID-19. As elsewhere in the world, these prevention measures have profoundly impacted Kenyan society and economy.

In particular, Kenyan culture is largely communal [7] where people depend on each other at all levels of society. The interdependence of individuals and society are inseparable and it is an inherent characteristic of Kenyan society with deep roots in its traditions. There is a profound sense of responsibility to others, and the extended family, clan, and community are foundational to the social structure and provide a sense of belonging [7]. In addition, religion is an integral part of most Kenyans and society with 85.5% of Kenyans identifying as Christians [8]. Many people go to places of worship for religious purposes and also to socialize with others such as family, and friends, and to meet new people. Thus, COVID-19 prevention measures not only had economic, social, and health consequences for all Kenyans, but the pandemic in the early stages disrupted social relationships and exacerbated inequalities [9] especially among people living with HIV (PLWH) who have additional unique challenges.

Despite improvements in HIV prevention and treatment, in 2021 Kenya was ranked 13^th^ in the world with a HIV/AIDS prevalence of 4.0 among adults aged 15–49 years [10]. According to the 2018 Kenya Population-based HIV Impact Assessment (KENPHIA) results, Kenya has a HIV prevalence of 4.9% (1.3 million people) for persons aged 15–64 years [11]. The prevalence among women is 6.6%, twice as much compared to men at 3.1%. Additionally, the HIV prevalence varies across counties. Wajir and Mandera counties in North Eastern Kenya have the lowest prevalence at 0.2% and Homa Bay county in Western Kenya has the highest prevalence of 19.6% [11]. Overall, HIV is a leading cause of mortality among adults [10], suggesting that a substantial share of the population may be adversely affected by COVID-19 prevention measures on HIV-related outcomes, particularly in Western Kenya where HIV prevalence is three times higher than the national estimate [11]. To mitigate health impacts, at the beginning of the pandemic in March 2020, the Kenyan Ministry of Health issued guidance for healthcare facilities to provide at least a three-month supply of antiretroviral (ARV) medication at each visit for all PLWH irrespective of viral load. This policy shift was designed to ensure patients had an extended supply of ARVs and to reduce the number of clinics visits needed [12]. However, the less studied social impacts of the pandemic, including the disruption of social networks and systems of support, could have important consequences for PLWH.

Studies have shown that social networks influence health behaviors and outcomes such as ARV adherence [13–15]. Social networks are a key avenue for providing and receiving social support. Social support includes the social resources that are provided, or are generally perceived to be available, from people in everyday formal and informal relationships. Social support includes emotional, informational, or instrumental (aid, tangible, material) resources that are provided or exchanged to promote health and well-being. Forms of social support vary depending on the situation [16]. For example, for a job loss, informational support could prove helpful by providing a resource for job advertisements, whereas emotional support might be most important when an individual is grieving the death of a loved one. Instrumental support could include babysitting for someone to attend an appointment or lending money to meet financial needs. All of these forms of social support have been shown to promote psychological and physical health [16]. Emotional support is an important type of social support [17,18] for wellbeing in many life situations because it reassures individual self-worth and it is an essential human need [19]. COVID-19 pandemic preventive measures, including physical distancing and restriction in movement, have impacted all forms of social support, which may have especially grave consequences among PLWH.

Social support has been shown to be a protective factor for the health of PLWH [20] and those with mental illness [21,22]. Disruption of social networks due to the mitigation efforts of COVID-19 could not only interrupt HIV care and exacerbate disease progression but erode emotional wellbeing and mental health [23,24]. Previous studies have reported that PLWH experience depression and anxiety disproportionately compared to people not living with HIV because of comorbidities and social issues, such as stigma [25–27]. Anxiety and depression among PLWH further impact morbidity and mortality due to opportunistic infections and loss of support [28–31]. However, the full impact of COVID-19 on the mental health and wellbeing of PLWH remains unclear.

The purpose of this study was to explore pandemic-related concerns among a cohort of PLWH in Kenya. Within this context, we also identify the demographic and social support factors associated with symptoms of depression and anxiety and measures of ARV adherence over the course of the pandemic.

## Methods

### Study setting

Patient data used in this study is drawn from participating sites in the East African region of the International Epidemiological Databases to Evaluate AIDS (EA-IeDEA) consortium. EA-IeDEA is one of seven regional networks making up the global IeDEA consortium which was established by the National Institute of Allergy and Infectious Diseases (NIAID) in 2005. The consortium allows for the collection and analysis of large cohorts of HIV/AIDS-related data [32–34]. The EA-IeDEA cohort includes patients from 84 clinics in Kenya, Tanzania, and Uganda which have been previously described [34]. This study took place in two IeDEA-affiliated programs: the *Academic Model Providing Access to Healthcare (AMPATH)* located in Eldoret, Kenya, and *Family AIDS Care and Education Services (FACES)* located in Kisumu, Kenya.

FACES is located in Kisumu County, the 13^th^-largest county in Kenya at the 2019 census [35]. Situated on the shores of Lake Victoria, Kisumu County has among the highest HIV prevalence in the nation at 17.5%. Economic activities in Kisumu include fishing in Lake Victoria and agriculture (rice, sugarcane, and subsistence farming) [36]. AMPATH clinics are located throughout western Kenya, with this study taking place in Uasin Gishu County, where HIV prevalence is 5.5%. Eldoret is the administrative center of Uasin Gishu County, which is the 11^th^-largest county in Kenya [35] and is the “breadbasket” of Kenya [37]. In addition, Eldoret is home to various economic activities such as large-scale maize, wheat, and dairy farming, and manufacturing.

### The “Syndemics” parent study

This study is part of a larger *“Syndemics*” cohort study examining the interlinked effects of alcohol, drug use, and mental health on HIV outcomes among newly diagnosed adults (≥18 years old) in Kenya and Uganda. A syndemic “is the presence of two or more disease states that adversely interact with each other, negatively affecting the mutual course of each disease trajectory, enhancing vulnerability, and which are made more deleterious by experienced inequities” [38]. Between January and October 2019, the S*yndemics* study recruited 576 PLWH from three clinical sites in Western Kenya and Uganda, including 200 from AMPATH in Eldoret and 200 from FACES in Kisumu. Eligibility for *Syndemics* included being at least 18 years old and not previously enrolled into HIV care. Eligible participants were provided with a written informed consent and completed the Client Diagnostic Questionnaire (CDQ) [39], a screening tool designed and validated for use with PLWH and to be administered by lay personnel with no formal mental health or substance use training. The CDQ evaluated symptoms of depression, anxiety, post-traumatic stress disorder, psychosis, and substance use disorders.

### The “Networks” sub-study

*Networks* was a sub-study of the larger *Syndemics* cohort study. *Networks* used a mixed methods social network approach to characterize the effects of substance use and mental health issues on engagement in care and HIV clinical outcomes among *Syndemics* participants from the AMPATH and FACES clinics in Kenya. Between November 2019 and March 2020, the *Networks* study collected egocentric network data from 174 people (88 from FACES in Kisumu; 86 from AMPATH in Eldoret). All 174 participants engaged in a brief survey and quantitative social networks questions, of whom 61 (n = 29 in Kisumu; n = 32 in Eldoret) were purposively sampled to answer additional open-ended questions about their networks. Eligibility was open to anyone enrolled in the larger *Syndemics* cohort study. In March 2020, the research team ended recruitment for the *Networks* study due to the global COVID-19 pandemic, including concerns for staff and participant safety.

### COVID-19 phone surveys

Participants enrolled in the EA-IeDEA *Syndemics* and *Networks* studies were contacted to participate in phone surveys about their experiences during the pandemic. The current analysis is restricted to the results of phone surveys conducted with participants in *Networks*.

From June—July 2020, during the initial wave of COVID-19, we contacted all *Networks* participants for an initial COVID phone survey; in total, we recruited 145 participants (75 from FACES in Kisumu and 70 from AMPATH in Eldoret). After obtaining verbal consent, due to COVID-19 pandemic, phone interviews were conducted to assess knowledge, health behaviors, sources of information, and illness experiences related to COVID-19, engagement in HIV care and treatment, changes to participants’ social networks and support systems, changes in mental health status and substance use, and key concerns and health needs in the context of the pandemic. The phone interviews lasted an average of 40 minutes and interviewers recorded all responses into a tablet programmed with Research Electronic Data Capture (RedCap) [40].

From May—June 2021, we conducted a second round of phone interviews. This was slightly over a year since the first COVID-19 case was reported in March 2020 and nearly all COVID-19 restrictions had been lifted, and vaccines had become available in March 2021 [41] although supplies were limited. In addition, as with other parts of the world, there was vaccine hesitancy [42]. In total, we recruited 130 *Networks* participants (67 from FACES in Kisumu; n = 63 from AMPATH in Eldoret). We repeated the initial questions to assess changes over time. Again, phone interviews lasted an average of 40 minutes. In total, 130 participants completed both surveys in 2020 and 2021 and are included in this analysis.

### Survey measures

Mental health status (past 2 weeks) was assessed by using the two brief screening tools: the Patient Health Questionnaire-2 (PHQ-2) [43] to measure current depression symptoms and the Generalized Anxiety Disorder 2-item (GAD-2) [44] to assess generalized anxiety disorder symptoms. The PHQ-2 has two questions that assess the number of days in the past 2 weeks the participant experienced depression symptoms. For the current study, the number of days the symptom was experienced was scored as follows: not at all (0), several days (1), more than half the days (2), nearly every day (3). A total depression score was obtained by summing individual item scores (PHQ-2 range = 0–6). The GAD-2 has two questions that assess the number of days in the past 2 weeks the participant experienced anxiety symptoms. The number of days the symptom was experienced was determined as follows: not at all (0 days), several days (1), more than half the days (2), nearly every day (3). A total anxiety score was obtained by summing individual item scores (GAD-2 range = 0–6). Scores of ≥3 out of 6 on PHQ-2 and GAD-2 subscales are considered symptomatic for depression and anxiety, respectively [43,44].

We assessed ARV adherence by combining the information from the following six questions: (1) Do you have problems taking your ARV medicines? (2) Have you forgotten to take your ARV medicines within the last 3 days? (3) Do you have problems taking your ARV medicines around others? (4) Have you taken a dose more than 1 hour late in the past 7 days? (5) Are there times when you are supposed to take the ARV medicine, but you don’t have them with you? (6) Have you missed at least one dose of ARV medicine in the past 7 days? If a participant answered yes to any of these six questions, then that was coded as “Yes.” If a participant answered “No” to all six questions, this was coded as a “No”.

The following six questions assessed forms of social support: (1) How has the amount of support you get for material things (money, housing, food, etc.) changed since the coronavirus pandemic started? (2) How has the amount of support you get for emotional or personal things changed since the coronavirus pandemic started? (3) How has the amount of support you get for HIV-related things changed since the coronavirus pandemic started? (4) How has the amount of support you get for other health-related things changed since the coronavirus pandemic started? (5) How has the amount of support you get for mental health-related things (like anxiety or depression) changed since the coronavirus pandemic started? (6) How has the amount of spiritual or religious support you get changed since the coronavirus pandemic started? The response categories to all these questions were: “I am getting more support”, “I am getting less support”, or “I am getting the same amount of support.” An additional question asked, “How has the support that you GIVE to your networks changed?” with the following response categories: “I am providing more support for people in my network”, “I am providing less support for people in my network”, or “I am providing the same amount of support for people in my network.”

Finally, we asked several open-ended questions about social support networks, including “Can you tell me generally how your interactions with people in your social network have changed since the pandemic?” and “Has the number of people you interact with gotten bigger, smaller, or stayed the same? Tell me a little bit more about that.” We also asked participants about their biggest concerns related to the coronavirus pandemic. Interviewers typed detailed notes of participant responses.

### Data analysis

COVID-19 phone survey data were merged with baseline *Networks* study data to obtain participant demographic characteristics. We used descriptive statistics to describe the sample. Bivariate analyses assess demographic and social support factors associated with symptoms of depression and anxiety, and problems related to ARV adherence. Quantitative analyses were conducted with SAS version 9.4 [45] and we used ∝ = 0.05 for the chi-square analysis.

Analysis of the qualitative survey data was guided by our quantitative findings. We exported the text data (i.e., short answers to open-ended survey questions typed up in participants’ own words) into Excel to content code, categorize, and sort participant answers based on our a priori questions. We first quantified the most frequently mentioned categories of concerns about the pandemic across both years. We next sorted the sample by how participants answered survey questions about their networks (network size, changes) and within each category, assessed how participants talked about their networks and forms of support to assess patterns. We then sorted participants by self-reported depression, anxiety, and adherence issues to assess the intersections with their social networks in terms of size, forms of support, and changes over the pandemic. In the quantitative data, we noted a statistically significant pattern that the small number of participants whose networks grew bigger and/or they received more support during the pandemic were also more likely to report mental health and adherence issues, and thus we examined participants’ network descriptions to explain these somewhat counterintuitive findings. Overall, our content analysis contextualizes the survey data, including the differences between how participants with mental health and adherence issues talked about their social networks during the pandemic compared to those who did not report these issues. Representative quotes were drawn and attributed only to the sex and age of participants to protect confidentiality.

### Ethics statement

All phases of research were overseen by the Institutional Review Boards or Ethics Committees of the participating sites: *AMPATH*: Moi University College of Health Sciences and Moi Teaching and Referral Hospital Institutional Research and Ethics Committee and *FACES*: Kenya Medical Research Institute/Scientific Ethics and Review Unit. In the United States, Institutional Review Boards at Indiana University and the University of California, Riverside also reviewed all protocols.

## Results

The current study involved 130 *Networks* participants who responded to both COVID-19 surveys in 2020 and 2021. We first compared the 130 participants included in the present analysis to the 44 eligible participants who were missing survey data; there were no statistically significant differences in terms of sex, age, education, marital status, and site (Table 1).

**Table 1:** Comparison of COVID-19 survey participants and nonparticipants, n = 174.

	COVID-19 survey participants in 2020 and 2021		
Demographics	COVID-19 survey n = 130 (74.7%)	No COVID-19 survey n = 44 (25.3%)	p-value
Sex			0.244
Male	48 (36.9)	12 (27.3)	
Female	82 (63.0)	32 (72.7)	
Age			0.929
18–34 years	59 (45.7)	20 (46.5)	
35+ years	70 (54.3)	23 (53.5)	
Education			0.526
Up to High school	91 (70.0)	33 (75.0)	
> High school	39 (30.0)	11 (25.0)	
Marital			0.185
Married	82 (63.6)	23 (52.3)	
Not married	47 (36.4)	21 (47.7)	
Site			0.662
FACES (Kisumu)	67 (51.5)	21 (47.7)	
AMPATH (Eldoret)	63 (48.5)	23 (52.3)	

Demographic characteristics for the 130 participants retained in the analysis are shown in Table 2. With an average age of 36.6 years, almost two-thirds (63%) were female, and over two-thirds (69%) had a primary or high school education. Over half (57%) were married and half (50%) were of Luo ethnicity. Consistent with the Kenyan economic environment, the majority of participants (80%) worked in the informal sector.

**Table 2:** Demographic Characteristics of Networks participants, n = 130.

Characteristic	n	Percent
Age groups, years		
18–24	15	11.6
25–34	44	34.1
35–44	36	27.9
45+ years	34	26.4
Mean, sd	129	36.6, 9.9 (20–62)
Sex		
Female	82	63.1
Male	48	36.9
Education		
None	1	0.7
Primary	46	35.4
High school	44	33.8
Vocational/college	30	23.1
University	9	6.9
Marital status		
Single	22	17.0
Married	73	56.6
Separated or divorced	12	9.3
Widowed	13	10.0
Common law or domestic partnership	9	7.0
Ethnicity		
Luo	64	50.4
Kalenjin	22	17.3
Luhya	25	19.7
Kikuyu	8	6.3
Other (Kisii, Turkana, Teso, Kamba)	8	6.3
Preferred spoken language		
English	10	7.7
Swahili	71	54.6
Kenyan Language–Luo	43	33.1
Other Kenyan language (Bukusu, Kalenjin, Kikuyu, Kisii)	4	3.1
Other language	2	1.5
Religion		
Christian	125	96.9
Muslim	3	2.3
None	1	0.8
Sexual orientation		
Heterosexual	128	98.5
Not sure/Undecided/In transition	2	1.5
Work sector*		
Formal	20	19.2
Informal	83	79.8
Prefer not to answer	1	1.0

*Question asked in 2020 COVID-19 survey only.

### Mental health concerns during the pandemic

Across the sample, we detected statistically significant increases in reported symptoms of anxiety and depression between 2020 to 2021 (Table 3). In 2020, near the beginning of the pandemic, higher proportions of participants reported no depression and anxiety symptoms compared to 2021, when we see increases in the overall PHQ-2 and GAD-2 positive scores, as well as an increased frequency of individual symptoms of depression and anxiety. These trends hold across both sites, though participants at FACES were more likely to report symptoms of depression (85% and 89%, p = 0.001 and p<0.0001 in 2020 and 2021 respectively). Moreover, we asked participants to give an overall subjective assessment of their mental health compared to their last visit. More than one-third said their mental health worsened in 2021 (from 24.2% to 36.4%), while a smaller percentage reported their overall mental health had improved (from 9.4% to 16.3%).

**Table 3:** 2020 and 2021 COVID-19 Surveys of mental health symptoms, (n = 130).

Mental health	First COVID survey June 2020		Second COVID survey May-June 2021		p-value***
	n	percent	n	percent	
Depression symptoms (PHQ-2)*					<0.0001
	20	15.4	56	43.1	
No	110	84.6	74	56.9	
Specific questions from PHQ-2					
Little interest or pleasure doing things					<0.0001
Not at all (0)	92	70.8	47	36.2	
Several days (1)	27	20.8	41	31.5	
More than half the days (2)	8	6.2	33	25.4	
Nearly every day (3)	3	2.3	9	6.9	
Feeling down, depressed or hopeless					<0.0001
Not at all (0)	71	54.6	35	26.9	
Several days (1)	34	26.2	45	34.6	
More than half the days (2)	17	13.1	41	31.5	
Nearly every day (3)	8	6.2	9	6.9	
Anxiety symptoms (GAD-2)**					<0.0001
Yes	18	13.8	43	33.1	
No	112	86.2	87	66.9	
Specific questions from GAD-2					
Feeling nervous, anxious					<0.0001
Not at all (0)	74	56.9	37	28.5	
Several days (1)	38	29.2	50	38.5	
More than half the days (2)	13	10.0	36	27.7	
Nearly every day (3)	5	3.8	7	5.4	
Not being able to stop or control worrying					0.0029
Not at all (0)	74	56.9	60	46.2	
Several days (1)	47	36.2	39	30.0	
More than half the days (2)	6	4.6	28	21.5	
Nearly every day (3)	3	2.3	3	2.3	
Overall mental health					0.0020
Same	85	66.4	61	47.3	
Worse	31	24.2	47	36.4	
Improved	12	9.4	21	16.3	

* The values of the two PHQ-2 specific questions were summed and a score of ≥ 3 suggests depression symptoms, reported as “Yes”.

**The values of the two GAD-2 specific questions were summed and a score of ≥ 3 suggests anxiety symptoms, reported as “Yes”.

***Signed Rank p-value.

To contextualize these survey questions, we examined our qualitative data about participants’ primary concerns during the pandemic. Concerns around infection and safety protocols were most commonly mentioned in both years by nearly half the sample. Slightly more participants in 2021 relayed a “fear” of contracting COVID-19, some of whom gave an ominous characterization of COVID-19 as an “unseen enemy” and expressed “fear of getting infected and the fact that it is difficult to tell who has it.” Overall, about 20% of the sample in 2021 expressed specific concerns around access to the vaccines that had been recently been developed, though several participants looked for broader answers:

*We should keep praying for God’s intervention to help us fight coronavirus*. [Female, 47 years]

*Praying that we shall have a lasting solution to the monster virus soon*. [Male, 55 years]

*The WHO [World Health Organization] should try so hard to find a permanent solution to coronavirus*. *The world is hurting*. [Female, 62 years]

Across 2020 and 2021, participants expressed other related concerns, including general social disruptions, economic crises and job loss, hunger and food insecurity, and concern for family members, including children. The precarity that the lockdown and restrictions had created in their lives and lingering uncertainty was frequently mentioned, as revealed in a sample of concerns listed in 2021:

*Not being able to meet my daily needs and now with restrictions and lockdown things are becoming even more difficult*. [Female, 30 years]

*The unavailability of work and food also has become scarce*. [Male, 36 years]

*Life has become unbearable due to tough economic times*. [Female, 38 years]

In both years, HIV was consistently, but less frequently, cited as a top concern by just under 10% of the sample in both surveys. HIV was interrelated with fear of increased susceptibility to acquiring coronavirus and concerns around access to vaccination, due to their immunocompromised status:

*My greatest concern is that those who are HIV infected are more at risk [for coronavirus] because of the compromised immunity*. [Male, 37 years]

*We are hoping that HIV patients and others with chronic illnesses like cancer will be given priority during public vaccination*. [Male, 30 years]

Taken together, these data suggest that PLWH participants shared multiple, interrelated, and ongoing health, economic, and social concerns about the pandemic, just as many other Kenyans share. However, these concerns could wear down the mental health of newly diagnosed HIV patients and potentially affect adherence as individuals are learning to manage their health condition.

### Mental health and social support

In 2020, we did not find any significant associations between mental health, ARV adherence, social support, and network characteristics. However, in 2021, we noted several significant trends in bivariate analyses, and thus the remainder of the results focus on these data. Overall, we note significant increases in some forms of support among those with mental health symptoms over the course of the pandemic.

As shown in Table 4, participants who were experiencing symptoms of depression were more likely to be married (p = 0.026) and from the FACES site in Kisumu (p < .0001). Depression symptoms were associated with reporting increases in several types of social support since the pandemic began, including material support (p = 0.008), emotional support (p = 0.01), HIV support (p = 0.001), other health-related support (p = 0.004), mental health-related support (p = 0.02), and spiritual support (p = 0.04).

**Table 4:** COVID-19 survey: Social support and demographic factors associated with depressive symptoms (PHQ-2*).

	Second COVID survey May-June 2021		
Characteristic	Depression symptoms n = 56 (43.1%)	No depression symptoms n = 74 (56.9%)	p-value
Network Interactions			0.64
Bigger	8 (14.5)	7 (9.5)	
Same	28 (50.9)	38 (51.4)	
Smaller	19 (34.5)	29 (39.2)	
Material support			0.0087^§^
More	8 (14.5)	3 (4.1)	
Same	5 (9.1)	20 (27.0)	
Less	42 (76.4)	51 (68.9)	
Emotional support			0.010^§^
More	8 (14.5)	3 (4.1)	
Same	8 (14.5)	25 (33.8)	
Less	39 (70.9)	46 (62.2)	
HIV support			0.0010^§^
More	10 (18.2)	3 (4.1)	
Same	9 (16.4)	31 (41.9)	
Less	36 (65.5)	40 (54.1)	
Health support			0.0027^§^
More	5 (9.1)	1 (1.4)	
Same	8 (14.5)	28 (37.8)	
Less	42 (76.4)	45 (60.8)	
Mental health support			0.0187^§^
More	7 (12.7)	3 (4.1)	
Same	10 (18.2)	28 (37.8)	
Less	38 (69.1)	43 (58.1)	
Spiritual support			0.035
More	9 (16.4)	6 (8.1)	
Same	10 (18.2)	28 (37.8)	
Less	36 (65.5)	40 (54.1)	
Change in support that you give			0.743^§^
More	4 (7.3)	3 (4.1)	
Same	13 (23.6)	18 (24.3)	
Less	38 (69.1)	53 (71.6)	
Sex			0.80
Male	20 (35.7)	28 (37.8)	
Female	36 (64.3)	46 (62.2)	
Education			0.22
Up to High school	36 (64.3)	55 (74.3)	
> High school	20 (35.7)	19 (25.7)	
Age			0.08
18–34 years	30 (54.6)	29 (39.2)	
35+ years	25 (45.4)	45 (60.8)	
Marital status			0.026
Married	41 (74.5)	41 (55.4)	
Not married	14 (25.5)	33 (44.6)	
Site			< .0001
FACES (Kisumu)	50 (89.3)	17 (23.0)	
AMPATH (Eldoret)	6 (10.7)	57 (77.0)	

^§^ Fishers Exact Test.

* Patient Health Questionnaire-2 (PHQ-2).

Table 5 shows similar associations between anxiety and social support. Individuals experiencing symptoms of anxiety reported receiving more of the following types of social support since the pandemic began: material support (p = 0.02), emotional support (p = 0.03), and mental health-related support (p = 0.02). Additionally, participants experiencing anxiety symptoms were more likely to be from the FACES site in Kisumu (p < .0001).

**Table 5:** COVID-19 survey: Social support and other factors associated with anxiety (GAD-2*).

	Second COVID survey May-June 2021		
Characteristic	Anxiety symptoms N = 43 (33.1%)	No anxiety symptoms N = 87 (66.9%)	p-value
Network Interactions			0.054
Bigger	9 (21.4)	6 (6.9)	
Same	19 (45.2)	47 (54.0)	
Smaller	14 (33.3)	34 (39.1)	
Material support			0.0202^§^
More	7 (16.7)	4 (4.6)	
Same	4 (9.5)	21 (24.1)	
Less	31 (73.8)	62 (71.3)	
Emotional support			0.045^§^
More	7 (16.7)	4 (4.6)	
Same	7 (16.7)	26 (29.9)	
Less	28 (66.7)	57 (65.5)	
HIV support			0.204
More	7 (16.7)	6 (6.9)	
Same	11 (26.2)	29 (33.3)	
Less	24 (57.1)	52 (59.8)	
Health support			0.995^§^
More	3 (7.1)	3 (3.4)	
Same	7 (16.7)	29 (33.3)	
Less	32 (76.2)	55 (63.2)	
Mental health support			0.026^§^
More	7 (16.7)	3 (3.4)	
Same	9 (21.4)	29 (33.3)	
Less	26 (61.9)	55 (63.2)	
Spiritual support			0.361
More	7 (16.7)	8 (9.2)	
Same	10 (23.8)	28 (32.2)	
Less	25 (59.5)	51 (58.6)	
Change in support that you give			0.84^§^
More	3 (7.1)	4 (4.6)	
Same	10 (23.8)	21 (24.1)	
Less	29 (69.0)	62 (71.3)	
Site			< .0001
FACES (Kisumu)	38 (88.4)	29 (33.3)	
AMPATH (Eldoret)	5 (11.6)	58 (66.7)	

^§^ Fisher’s Exact Test.

* Generalized Anxiety Disorder 2-item (GAD-2).

Looking to the qualitative data indicated that forms of support are related to network size and shifting relationships during the pandemic, explored further below.

### Network size and shifting support during the pandemic

About half of all participants (51%) reported that the size of their networks *remained the same* during the pandemic. When probed to talk more about how, if at all, their networks had changed, most of these participants reported that their social networks primarily consisted of family and close ties that endured through the challenges:

*I have maintained the same people in my network circle because we have been solid and supporting each other where need be*. [Female, 50 years]

*It has stayed the same because the majority are family members and other relatives*. [Male, 45 years]

*Just maintained the friends I had because they have been true friends and always there for me whenever I have a problem*. [Female, 39 years]

However, among those who said the *size* of their network had not changed, nearly 20% reported that the *forms of support* that they give or receive had decreased due to the pandemic:

*The people in my network are still the same people*, *interaction has remained the same*, *but with less material support [due to job loss]*. [Female, 51 years]

The people I interact with have remained the same but with very less support compared to the last survey. [Female, 36 years]

In total, 37% of the sample reported that their networks became *smaller* during the pandemic. Reasons for network shrinkage included people relocating, economic circumstances and job loss, and that many people were struggling to care for their own families, having less regular communication with their networks and offering less forms of support. These circumstances led many to become more insular during the pandemic:

*Everyone is struggling for their own needs and that of their family and with coronavirus worsening things*, *interactions have become so rare*. [Female, 33 years]

*I had to cut the number of people we are interacting with because I was not gaining from them and also I was not offering support to them since I am economically down compared to before COVID*. [Female, 27 years]

*The majority are also struggling as I do and therefore they are more concerned with their families and life and thus can’t find time to interact or support me*. [Female, 26 years]

However, 12% (n = 15) reported that the size of their network *increased* in 2021. Of these, more than half (n = 8) reported depression and/or anxiety symptoms. Perhaps initially counterintuitive, qualitative data and field observations suggest that people already embedded in strong networks were able to mobilize their social support ties and build more connections during a time of crisis, especially in regard to coping with their anxiety and depression. This may help explain the positive association between mental health symptoms and receiving more forms of social support, as observed in the quantitative data:

*I have added more people in my network so that we can support each other emotionally and spiritually*. [Male, 31 years]

*I have added more people in my network circle*, *specifically church members who have been so supportive during this difficult moment [of the] covid-19 pandemic* [Male, 47 years]

*I have more people in my network circle and that’s encouraging since we support each other as much as we can*. *To me unity is strength*. [Female, 47 years]

### ARV adherence and social support

Table 6 shows associations between reporting problems with ARV adherence and social support. Individuals experiencing challenges with ARV adherence reported receiving more mental health-related social support (p = 0.016) and other health-related social support (p = 0.009). Furthermore, participants experiencing ARV adherence challenges were more likely to be married (p = 0.03) and from the AMPATH site in Eldoret (p < .0001).

**Table 6:** COVID-19 survey: Social support and other factors associated with ARV* Problems.

	Second COVID survey May-June 2021		
Characteristic	Problems adhering N = 64 (49.2%)	No Problems N = 66 (50.7%)	p-value
Network Interactions			0.16
Bigger	7 (10.9)	8 (12.3)	
Same	28 (43.7)	38 (58.5)	
Smaller	29 (45.3)	19 (29.2)	
Material support			0.122
More	5 (7.8)	6 (9.2)	
Same	17 (26.6)	8 (12.3)	
Less	42 (65.6)	51 (78.5)	
Emotional support			0.056
More	6 (9.4)	5 (7.7)	
Same	22 (34.4)	11 (16.9)	
Less	36 (56.2)	49 (75.4)	
HIV support			0.133
More	5 (7.8)	8 (12.3)	
Same	25 (39.1)	15 (23.1)	
Less	34 (53.1)	42 (64.6)	
Health support			0.007^§^
More	1 (1.6)	5 (7.7)	
Same	25 (39.1)	11 (16.9)	
Less	38 (59.4)	49 (75.4)	
Mental health support			0.015^§^
More	3 (4.7)	7 (10.8)	
Same	26 (40.6)	12 (18.5)	
Less	35 (54.7)	46 (70.8)	
Spiritual support			0.09
More	5 (7.8)	10 (15.4)	
Same	24 (37.5)	14 (21.5)	
Less	35 (54.7)	41 (63.1)	
Change in support that you give			0.312^§^
More	3 (4.7)	4 (6.1)	
Same	19 (29.7)	12 (18.5)	
Less	42 (65.6)	49 (75.4)	
Marital			0.027
Married	34 (53.9)	48 (72.7)	
Not married	29 (46.0)	18 (2.3)	
Site			< .0001
FACES	21 (32.8)	46 (69.7)	
AMPATH	43 (67.2)	20 (30.3)	

^§^ Fisher’s Exact Test.

* ARV problems were assessed by combining the information from the following six ARV problems questions: 1) Do you have problems taking your ARV medicines? 2) Have you forgotten to take your ARV medicines within the last 3 days? 3) Do you have problems taking your ARV medicines around others? 4) Have you taken a dose more than 1 hour late in the past 7days? 5) Are there times when you are supposed to take the ARV medicine but you don’t have them with you? 6) Have you missed at least one dose of ARV medicine in the past 7 days? If a participant answered yes to any one of these six questions, then that was coded as “Yes”. If a participant answered “No” to all these six questions, this was coded as a “No”.

The open-ended responses about network size and quality of interactions also lend insight into the ARV survey data. Most participants who reported adherence problems across both sites reported receiving less social support over the course of the pandemic and said their support remained the same compared to those without adherence issues.

However, we found a small number of participants at both sites who reported receiving *more* support. At FACES, where fewer overall participants reported problems with ARV adherence, four also reported depression and anxiety symptoms and are represented in the section above among those reaching out to create bigger networks to mobilize support for themselves. We found a similar trend at AMPATH, where three participants (who did not report any mental health symptoms) said their network size increased due to cultivating closer relations with family and colleagues:

*I have acquired new friends*, *I have been able to disclose my [HIV] status to more people*, *including my sisters*, *and now I am able to receive more support from them*. [Female, 23 years]

On the other hand, several others from AMPATH discussed their HIV status as a reason for receiving *less* support. Two women said they were stigmatized by their network since their diagnosis and two others reported that their network members perceived their health status as improving since enrolling in care, and therefore they required less support:

*People were closer and supportive of me in the past because I have been sick and not able to provide for myself and my family but now that I have regained my health*, *I rarely receive support from my networks*. [Female, 37 years]

*Interactions have become very rare with people nowadays*, *they don’t care so much about me because they think I am okay and not so sick as before*. [Male, 44 years]

## Discussion

Our study explored pandemic-related experiences among a cohort of PLWH in Kenya. Within this context, we also examined the demographic and social support factors associated with symptoms of depression and anxiety and measures of ARV adherence during the first two years of the pandemic among a group of PLWH newly enrolled into care in two IeDEA clinics in Kenya. Participants reported a variety of concerns over the course of the pandemic and we documented statistically significant increases in symptoms of depression and anxiety, which also affected some participants’ ability to adhere to their ARV medication. Overall, the size and quality of participants’ network interactions and forms of support shifted during the pandemic, and we detected small but significant differences in the ways that social networks may shape patient outcomes. Our study has implications for HIV care and ensuring patient health and wellbeing, particularly amidst the challenges of the ongoing global COVID-19 pandemic.

First, it is important to note that participants reported multiple and interrelated health, social, and economic concerns across both surveys, in addition to their HIV status that rendered them at heightened risk of infection with COVID-19. These findings reflect surveys conducted by the World Bank which found that Kenyans were anxious about the pandemic in 2020, and remained anxious in 2021 regarding fear of infection, job losses, and economic crisis [46]. Given the ongoing uncertainty of the pandemic, the role of social networks and ability to access forms of support may be key strategies for coping that can be integrated into clinical care.

In general, social support has been shown to have positive effects on mental health and physical wellbeing, both by acting as a buffer during times of stress and as a main effect even in times of low stress [47]. Specifically, and as demonstrated by our qualitative findings, integration in a large social network can provide support to withstand troubling economic times and increase positive affect [47]. Our findings are consistent with other research showing that social support has buffered the impact of the COVID-19 pandemic on depression and anxiety [48]. In addition, studies among PLWH have shown that religious activities offer a buffer against stressful life events [49,50], which is reflected in our findings on spiritual support.

The global pandemic refashioned participants’ networks and often led to changes in support. Even if the size of networks remained consistent across the 2020 and 2021 pandemic years (the pandemic was still ongoing at the time of writing), often the quality and frequency of interactions and forms of support weakened, as individuals struggled to navigate shifting social and economic conditions.

Importantly, however, we found that a small group of participants reached out to their networks to expand their contacts and mobilize support in the context of experiencing symptoms of depression and anxiety and/or when having difficulties adhering to ARVs. Mobilization of social support has been identified as a coping strategy during times of stress (e.g., natural disasters, illness). Mobilization may result from a person proactively seeking support during times of stress, or from one’s network responding to the onset of illness [51] (in this case, symptoms of anxiety or depression or trouble adhering to ARV regimens). In either case, both perceptions of support availability and realized support are key to coping and recovering from stressful events.

We also note significant site differences in terms of both mental health symptoms (reported more frequently at FACES) and issues with ARV adherence (reported more frequently at AMPATH). While these site differences could be an artifact of data collection, other social and political factors could be at play. In terms of ARV adherence, the AMPATH clinical catchment area had a high number of cases early on in the pandemic, prompting many people to avoid towns and hospitals, which could explain less adherence at that site. In contrast, FACES developed a proactive COVID-19 response, immediately reaching out to patients and implementing early medication refills and extra 3-month supplies of ARVs to mitigate adherence and retention lapses [52]. This could also have been due to structural factors such as ARV shortages [53]. Site differences in mental health are also challenging to interpret. One plausible explanation is that Eldoret (where AMPATH is located) is more cosmopolitan than Kisumu, and it could be that participants have fewer close relatives and forms of support within close proximity even in non-pandemic times, and thus their mental health was less directly affected compared to those in Kisumu.

More broadly, the underlying sociopolitical complexities in Western Kenya could also have differential but tangible impacts on patient healthcare and mental health outcomes across sites. The entire western region of Kenya has experienced post-election violence [54,55] and at the time of the study was living with the uncertainty of the elections in 2022, which people worried could affect social relations and mental health status because of increased political activities and tensions. These tensions could be exacerbated by pandemic-induced economic stressors, restrictions on mobility and/or increased out-migration to rural areas, and related concerns over access to resources and daily living essentials that may play out differently in these communities. We are unable to disentangle these complexities in a survey alone, and recommend further investigation with in-depth qualitative methods to assess how social and structural factors shape mental health and engagement in HIV care across time.

Our study has strengths and limitations. Phone-based surveys conducted during a pandemic are limited in their ability to capture the complexity of people’s everyday lives. This limitation is especially salient in terms of brief mental health assessments, as in-person interviews reveal additional dynamics difficult to detect via phone, including posture, gait, and physical appearance that could lend additional insight into participant affect. We acknowledge that our small sample sizes at each site precluded us from multivariate analyses, and there may be confounding factors in our bivariate analysis. Nonetheless, we detected several statistically significant associations and adding open-ended questions to our quantitative tool provided critical context about network size and quality of social support, including changes over time that otherwise would have been missed. Although some of our numbers are small, our findings generate hypotheses regarding the importance of mobilizing support in times of crisis that could be tested in future research and leveraged in health interventions.

Taken together, our study underscores the important role that social networks play in the physical and mental health and wellbeing of PLWH. While HIV was part of participants’ concerns and shaped their network relations in key ways, living with HIV was intertwined with other experiences related to the pandemic. Our findings call for holistic approaches to HIV care that consider the broader political, economic, and social contexts that shape its effectiveness.
