# Correction: Social support and the effects of the COVID-19 pandemic among a cohort of People Living with HIV (PLWH) in Western Kenya

**DOI:** 10.1371/journal.pgph.0005110

**Published:** 2025-08-21

**Authors:** Adel Mburia-Mwalili, Karla D. Wagner, Edith Kamaru Kwobah, Lukoye Atwoli, Maurice Aluda, Brianna Simmons, Jayne Lewis-Kulzer, Suzanne Goodrich, Kara Wools-Kaloustian, Jennifer L. Syvertsen

The Data Availability statement for this article is incorrect. The correct statement is: The data cannot be made publicly available due to ethical restrictions. Data can be made available due interested researchers by contacting the IeDEA project, the Custodian of the data. Kara Wools-Kaloustian is the PI for the EA IeDEA and is the direct contact for the data. Below is her information:

Kara Wools-Kaloustian, MD

Email: Kwools@iu.edu

School of Medicine

Indiana University
